# Differences in lifestyle behaviors, dietary habits, and familial factors among normal-weight, overweight, and obese Chinese children and adolescents

**DOI:** 10.1186/1479-5868-9-120

**Published:** 2012-10-02

**Authors:** Xiaofan Guo, Liqiang Zheng, Yang Li, Shasha Yu, Guozhe Sun, Hongmei Yang, Xinghu Zhou, Xingang Zhang, Zhaoqing Sun, Yingxian Sun

**Affiliations:** 1 Department of Cardiology, the First Hospital of China Medical University, 155 Nanjing North Street, Heping District, Shenyang, 110001, People’s Republic of China; 2 Department of Clinical epidemiology, Library, Shengjing Hospital of China Medical University, Shenyang, People’s Republic of China; 3 Department of Cardiology, Shengjing Hospital of China Medical University, Shenyang, People’s Republic of China

**Keywords:** Overweight, Obesity, Children, Adolescents, Health-related factors

## Abstract

**Background:**

Pediatric obesity has become a global public health problem. Data on the lifestyle behaviors, dietary habits, and familial factors of overweight and obese children and adolescents are limited. The present study aims to compare health-related factors among normal-weight, overweight, and obese Chinese children and adolescents.

**Methods:**

We conducted a cross-sectional study consisted of 4262 children and adolescents aged 5–18 years old from rural areas of the northeast China. Anthropometric measurements and self-reported information on health-related variables, such as physical activities, sleep duration, dietary habits, family income, and recognition of weight status from the views of both children and parents, were collected by trained personnel.

**Results:**

The prevalence rates of overweight and obesity were 15.3 and 6.4%, respectively. Compared to girls, boys were more commonly overweight (17.5% vs. 12.9%) and obese (9.5% vs. 3.1%). Approximately half of the parents with an overweight or obese child reported that they failed to recognize their child’s excess weight status, and 65% of patients with an overweight child reported that they would not take measures to decrease their child’s body weight. Obese children and adolescents were more likely to be nonsnackers [odds ratio (OR): 1.348; 95% confidence interval (CI): 1.039–1.748] and to have a family income of 2000 CNY or more per month (OR: 1.442; 95% CI: 1.045–1.99) and less likely to sleep longer (≥7.5 h) (OR: 0.475; 95% CI: 0.31–0.728) than the normal-weight participants.

**Conclusions:**

Our study revealed a high prevalence of overweight and obesity in a large Chinese pediatric population. Differences in sleep duration, snacking, family income, and parental recognition of children’s weight status among participants in different weight categories were observed, which should be considered when planning prevention and treatment programs for pediatric obesity.

## Background

The worldwide prevalence of pediatric overweight and obesity has increased dramatically in recent years. It was reported that in 2010, 43 million children were estimated to be overweight and obese, and 92 million were at risk of becoming overweight. The worldwide prevalence of childhood overweight and obesity increased from 4.2% in 1990 to 6.7% in 2010 [1]. A high prevalence and increasing trend have been observed in many parts of the world [2-6]. In the most developed metropolis of China, the prevalence of overweight in children increased 2-3–fold between 1985 and 1995 [6]. Childhood obesity has become a global public health problem that has raised concern worldwide [7,8].

The associated health risks and health care costs of childhood and adolescent obesity are considerable, the consequences of which include metabolic disorders, earlier puberty and menarche in girls, type 2 diabetes, hypertension, sleep apnea, adulthood obesity, and higher rates of mortality in young adults [8-13]. Given the huge health burden resulting from pediatric overweight and obesity, efforts should be made to prevent the onset of overweight/obesity and its associated diseases during early childhood.

Overweight and obesity among children and adolescents are likely to be the result of complex interactions between genes, lifestyle behaviors, dietary habits, and socioeconomic factors. As the targets of many public health strategies, life-related factors are modifiable, and they have been highlighted in many investigations. It is evidenced that life-related factors, such as physical activity, eating habits, and family income, are associated with pediatric overweight and obesity [7,8,14,15].

As the management of overweight/obesity among children and adolescents is often based on lifestyle modifications, recognition of the differences in lifestyle behaviors, dietary habits, or familial factors among children and adolescents with different weight status is the first step to take. However, the results of studies varied markedly due to differences in variables such as region and race. In rural areas of the northeast China, the health resources are relatively poor, the situation is often ignored by many health workers, and related data among children and adolescents, such as dietary habits and anthropological measurement results, are lacking. Therefore, we performed this study to measure 1) the prevalence of overweight and obesity in a large population sample of children and adolescents in the northeast China and 2) the differences in lifestyle behaviors, dietary habits, and familial factors in the participants with different weight status.

## Methods

### Study population

**Figure 1:**
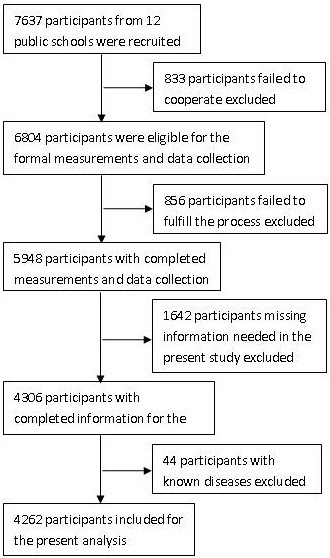
Flow chart of participant recruitment and derivation.

We conducted a cross-sectional study from July 2010 to January 2011 in rural areas of Shenyang, Liaoning Province, aiming to assess the differences in lifestyle behaviors, dietary habits, and familial factors among children and adolescents with different weight status. A total of 7637 students aged 5–18 years old were recruited. We adopted a multistage, stratified, cluster-sampling scheme and included participants from the northern, southern, western, and eastern regions of rural Shenyang. Three public schools were selected randomly from each geographic region. Samples from all of the classes in each school were included. In total, 12 public schools and 162 classes from these regions were selected. The overall response rate was 89%. Students known to have chronic heart, renal, or hepatic disease were excluded. The final sample included 4262 students (2196 boys and 2066 girls). Figure 1 shows the process of recruitment and derivation of the population. Informed consent was obtained from the parents of all subjects. The study was approved by the Ethics Committee of China Medical University.

### Data collection

Information on personal characteristics, including age, gender, race, physical activities (e.g., jogging) and sedentary behaviors (e.g., watching television), transportation mode to school, sleep duration (including nocturnal sleep duration and nap duration), smoking and drinking status, parental education level, and family income, was collected by trained personnel (cardiologists, doctors of internal medicine, and pediatricians) using a questionnaire. The frequency of having breakfast and key food items (such as fresh fruit and vegetables) was evaluated. The possible responses were “never or occasionally,” “less than once a week,” “1–3 days per week,” “4–6 days per week,” and “everyday.” The students were asked to rate their own level of weight status using the statements “too thin,” “about right,” “a little fat,” or “too fat.” Each participant was also asked to choose whether he/she would “put on weight,” “lose weight,” “stay the same as present,” or “do nothing to his/her weight status” in the future. The same questions were answered by their parents to assess the parents’ attitudes and any steps they to take regarding their children’s weight status. The content validity of the questionnaire was assessed by specialists of the related field. Seven specialists were invited to evaluate the representativeness of each item against the aim of the questionnaire. A score for each item was required (1 = no correlation; 2 = weak correlation; 3 = strong correlation; 4 = extreme correlation). A high content validity index of 0.89 basing on the number of the specialists and their scores was observed, indicating a good content validity. Intraclass correlation coefficients were used to examine the test-retest agreement. Five hundred participants were selected randomly to complete the questionnaire a second time one month after the initial interview. The 1-month test-retest agreement for the questionnaire was 0.81. Internal reliability was tested by Cronbach's alpha, and a value of 0.848 was obtained, indicating a good consistency of the questionnaire.

### Anthropometric measurements

Blood pressure (BP) was measured by trained personnel using a mercury sphygmomanometer and an appropriately sized cuff after each subject had rested for at least 5 min. The participants were advised to avoid coffee, tea, and exercise for at least 30 min before the measurement, and they remained seated with their arms supported at the level of the heart during the measurement. The average of two measurements was used in the analysis. With the subjects wearing light clothes without shoes, body weight was measured to the nearest 100 g by using a professional scale, and height was measured to the nearest 0.5 cm by using a stadiometer. Body mass index (BMI) was calculated using the formula weight (kg)/height^2^ (m^2^).

BP status was defined according to the Fourth Report on the Diagnosis, Evaluation, and Treatment of High Blood Pressure in Children and Adolescents [16]. Pre-hypertension was defined as a systolic BP (SBP) and/or diastolic BP (DBP) ≥90th percentile and <95th percentile for age, gender and height, or alternatively, by an SBP of more than 120 mmHg or a DBP of more than 80 mmHg. Hypertension was defined as an SBP and/or DBP ≥95th percentile. Weight class was defined according to the tables of the International Obesity Task Force based on data from the U.S., Brazil, The Netherlands, Hong Kong, United Kingdom, and Singapore [17].

### Statistical analysis

Continuous variables were expressed as mean values and standard deviation (SD), whereas categorical variables were described as frequencies and percentages. Chi-square analyses were used to examine associations between the categorical variables and weight status. Continuous variables were compared between different weight status by using one-way analyses of variance. The association between overweight or obesity and health-related factors was tested using multivariable logistic regression models, with odds ratios (ORs) and 95% confidence intervals (CIs) calculated. All statistical analyses were performed using SPSS version 17.0 software, and *P* < 0.05 indicated statistical significance.

## Results

The study sample consisted of 2196 boys and 2066 girls with a mean age of 11.04 ± 2.7 years. Of the 4262 participants, 15.3% were overweight, and 6.4% were obese. The prevalence of obesity was 9.5 and 3.1% among male and female participants, respectively. Overweight was found in 384 boys (17.5%) and 267 girls (12.9%). The prevalence of obesity decreased with age (5–8-year-olds: 7.6%; 9–11-year-olds: 7.2%; 12–14-year-olds: 5.9%; 15–18-year-olds: 3.1%).

**Figure 2:**
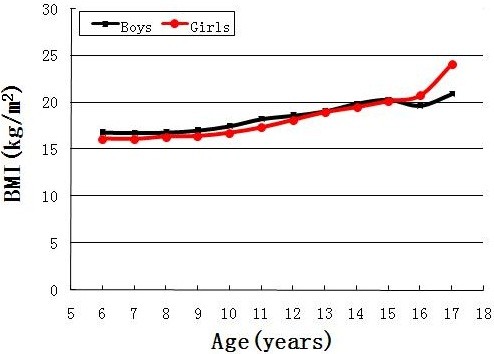
**Median body mass index by age and gender.** The 25^th^ and 75^th^ percentiles of BMI were 16.3 and 20.5 kg/m^2^, respectively.

**Figure 3:**
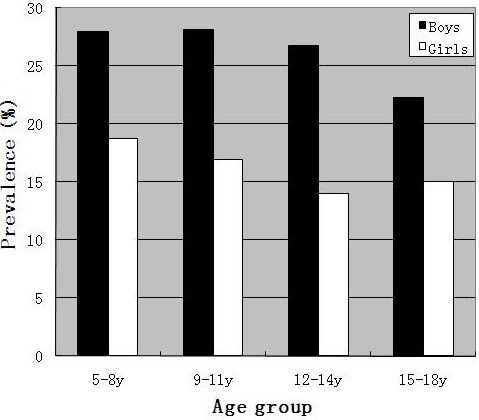
Prevalence of overweight and obesity by age and gender.

Figure 2 shows the median BMI by age and gender. The median BMI for boys was close to that of girls before the age of 15. The median BMI among girls (24.1 kg/m^2^) was higher than that of their male counterparts (21.0 kg/m^2^) at the age of 17. Figure 3 presents the prevalence of overweight plus obesity in different age groups by gender. Approximately one-third of boys and one-fifth of girls aged 5–11 years old had excess weight. Boys had higher prevalence than girls in all of the age categories.

Table 1 presents the baseline characteristics of the participants according to BMI categories. Boys had much higher proportions of overweight and obese participants. The Han Race predominated in all three weight classes. Hypertension was more common among overweight and obese participants than among those with normal BMI.

**Table 1:** Baseline characteristics of the participants by weight status (N = 4262)

	**Normal (n = 3338)**	**Overweight (n = 651)**	**Obese (n = 273)**	**Total (N = 4262)**	***P*-value**
**Gender**					<0.001^*^
Boys	1603(48)	384(59)	209(76.6)	2196(51.5)	
Girls	1735(52)	267(41)	64(23.4)	2066(48.5)	
**Age**					0.035^*^
5-8y	676(20.3)	140(21.5)	67(24.5)	883(20.7)	
9-11y	1148(34.4)	231(35.5)	107(39.2)	1486(34.9)	
12-14y	1152(34.5)	211(32.4)	85(31.1)	1448(34)	
15-18y	362(10.8)	69(10.6)	14(5.1)	445(10.4)	
**Race**					0.197
Han	2762(82.7)	524(80.5)	212(77.7)	3498(82.1)	
Manchu	323(9.7)	75(11.5)	37(13.6)	435(10.2)	
Mongol	122(3.7)	26(4)	15(5.5)	163(3.8)	
Others	131(3.9)	26(4)	9(3.3)	166(3.9)	
**Weight(kg)**	37.11 ± 10.8	49.18 ± 14.63	60.66 ± 18.44	40.46 ± 13.86	<0.001^*^
**Height(cm)**	143.79 ± 14.77	145.88 ± 15.1	146.52 ± 16	144.28 ± 14.93	<0.001^*^
**BMI(kg/m**^**2**^**)**	17.5 ± 2.1	22.4 ± 2.6	27.5 ± 3.9	18.9 ± 3.7	<0.001^*^
**BP status**					<0.001^*^
Normal	2304(69)	335(51.5)	99(36.3)	2738(64.2)	
Prehypertension	492(14.7)	116(17.8)	44(16.1)	652(15.3)	
Hypertension	542(16.2)	200(30.7)	130(47.6)	872(20.5)	

Data are expressed as n (%) or as the mean ± SD. *BMI*: body mass index; *BP*: blood pressure.

^*^P < 0.05, statistically significant difference.

Tables 2, 3, 4 present the differences in life-related factors among normal-weight, overweight, and obese participants. More children and adolescents with normal weight self-reported the consumption of snacks ≥4 days/week compared to their obese counterparts (22.7% vs. 16.5%, *P* < 0.05). A higher self-reported family income (≥2000 CNY/month) was more common among obese participants (82.1%) than among those with normal weight (75.9%). More overweight and obese participants were found to have a small family size (total member ≤3). No significant difference was observed in other self-reported variables such as sleep duration, physical activity, and the consumption of dietary products among the three weight categories.

**Table 2:** Differences in lifestyle behaviours among the participants with different weight status

	**Normal (n = 3338)**	**Overweight (n = 651)**	**Obese (n = 273)**	***P*-value**	
				**Overweight vs. normal**	**Obesity vs. normal**
**Sleep duration(h/day)**				0.169	0.089
<8	370(11.1)	62(9.5)	37(13.6)		
8-9	1104(33.1)	238(36.6)	102(37.4)		
≥9	1864(55.8)	351(53.9)	134(49.1)		
**Physical activity(times/week)**				0.34	0.215
None	91(2.7)	17(2.6)	4(1.5)		
1 to 3	1774(53.1)	331(50.8)	140(51.3)		
>3	1473(44.1)	303(46.5)	129(47.3)		
**Watching TV ≥3 h/day**	617(18.5)	116(17.8)	61(22.3)	0.688	0.116
**Playing video games and using computers ≥3 h/day**					
	220(6.6)	43(6.6)	14(5.1)	0.989	0.345
**Walking to school**	1780(53.3)	346(53.1)	134(49.1)	0.934	0.177
**Nonsmoking**	2880(86.3)	553(84.9)	224(82.1)	0.369	0.053
**No alcohol use**	2810(84.2)	545(83.7)	223(81.7)	0.767	0.279

Data are expressed as n (%).

**Table 3:** Differences in dietary characteristics among the participants with different weight status

	**Normal (n = 3338)**	**Overweight (n = 651)**	**Obese (n = 273)**	***P*-value**	
				**Overweight vs. normal**	**Obesity vs. normal**
**Having breakfast**				0.606	0.662
Every day	2332(69.9)	448(68.8)	186(68.1)		
1–6 days/week	336(10.1)	74(11.4)	26(9.5)		
Occasional or none	670(20.1)	129(19.8)	61(22.3)		
**Fresh fruit ≥4 days/week**	2371(71.1)	484(74.3)	181(66.3)	0.086	0.099
**Vegetables ≥4 days/week**	2696(80.8)	537(82.5)	222(81.4)	0.305	0.824
**Milk ≥4 days/week**	1659(49.7)	304(46.7)	123(45.1)	0.161	0.14
**Snacks ≥4 days/week**	757(22.7)	143(22)	45(16.5)	0.691	0.018^*^
**Fizzy drinks ≥4 days/week**	412(12.3)	79(12.1)	27(9.9)	0.883	0.233
**Fastfood ≥4 days/week**	156(4.7)	29(4.5)	12(4.4)	0.808	0.834

Data are expressed as n (%). ^*^P < 0.05, statistically significant difference.

**Table 4:** Differences in familial factors among the participants with different weight status

	**Normal (n = 3338)**	**Overweight (n = 651)**	**Obese (n = 273)**	***P*-value**	
				**Overweight vs. normal**	**Obesity vs. normal**
**Family income ≥2000 CNY/month**	2535(75.9)	500(76.8)	224(82.1)	0.637	0.022^*^
**Family size ≤ 3**	1806(54.1)	396(60.8)	165(60.4)	0.002^*^	0.043^*^
**Smoking mother**	230(6.9)	35(5.4)	19(7)	0.156	0.965
**Smoking father**	1705(51.1)	352(54.1)	152(55.7)	0.162	0.144
**Educational level of parents**				0.139	0.337
Both ≤ high school	2974(89.1)	568(87.3)	240(87.9)		
At least one > high school	189(5.7)	50(7.7)	21(7.7)		
Both > high school	175(5.2)	33(5.1)	12(4.4)		
**Unemployed mother**	605(18.1)	109(16.7)	50(18.3)	0.4	0.937
**Unemployed father**	283(8.5)	54(8.3)	23(8.4)	0.878	0.976

Data are expressed as n (%). ^*^P < 0.05, statistically significant difference. CNY: China Yuan. 1CNY = 0.157 USD.

Table 5 presents the recognition of weight status from the views of children and parents. Approximately one-fifth of the obese children thought they were “about right” and would “stay the same as present.” Among the parents of normal-weight children, 19.2% thought they were “too thin,” and 19.7% reported that they would take measures to increase the weight of their children. Approximately half of the parents with an overweight or obese child failed to recognize their child’s excess weight status.

**Table 5:** Differences in recognitions from children and parents towards the weight status

	**Normal (n = 3338)**	**Overweight (n = 651)**	**Obese (n = 273)**	***P*-value**	
				**Overweight vs. normal**	**Obesity vs. normal**
**Children**					
Attitude towards their weight status				<0.001	<0.001
Too thin	444(13.3)	11(1.7)	4(1.5)		
About right	2361(70.7)	235(36.1)	45(16.5)		
A little fat	497(14.9)	333(51.2)	137(50.2)		
Too fat	36(1.1)	72(11.1)	87(31.9)		
What will do to their weight status				<0.001	<0.001
should put on weight	508(15.2)	20(3.1)	3(1.1)		
should lose weight	418(12.5)	303(46.5)	189(69.2)		
stay the same as present	1827(54.7)	223(34.3)	49(17.9)		
no measures	585(17.5)	105(16.1)	32(11.7)		
**Parents**					
Attitude towards their weight status				<0.001	<0.001
Too thin	641(19.2)	20(3.1)	7(2.6)		
About right	2350(70.4)	262(40.2)	36(13.2)		
A little fat	321(9.6)	296(45.5)	120(44)		
Too fat	26(0.8)	73(11.2)	110(40.3)		
What will do to children's weight status				<0.001	<0.001
should put on weight	656(19.7)	29(4.5)	7(2.6)		
should lose weight	217(6.5)	235(36.1)	181(66.3)		
stay the same as present	1709(51.2)	246(37.8)	43(15.8)		
no measures	756(22.6)	141(21.7)	42(15.4)		

Data are expressed as n (%).

Table 6 presents the association between health-related factors and weight status according to multivariable logistic regression analysis. Overweight and obese participants were less likely to have a normal BP (OR: 0.437, 95% CI: 0.366–0.522 for overweight; OR: 0.206, 95% CI: 0.157–0.269 for obese). Obese children and adolescents were more likely to never or occasionally have snacks (OR: 1.348, 95% CI: 1.039–1.748) and to have a family income of 2000 CNY or more per month (OR: 1.442, 95% CI: 1.045–1.99) than those with normal weight. Normal-weight participants were significantly more likely to have long sleep duration than the obese participants and less likely to have a small family size than the overweight participants.

**Table 6:** Association between weight status and health-related factors

	**Normal**	**Overweight**			**Obese**		
	**OR (referent)**	**OR**	**95% CI**	***P*-value**	**OR**	**95% CI**	***P*-value**
Normal BP	1	0.437	0.366-0.522	<0.001^*^	0.206	0.157-0.269	<0.001^*^
Sleep duration ≥7.5 h	1	1.065	0.77-1.472	0.704	0.475	0.31-0.728	0.001^*^
Physical activity >3 times/week	1	1.098	0.927-1.3	0.28	1.12	0.872-1.438	0.375
Watching TV ≥3 h/day	1	0.93	0.747-1.159	0.521	1.191	0.881-1.611	0.256
Playing video games and using computers ≥3 h/day	1	0.963	0.685-1.353	0.828	0.699	0.399-1.224	0.211
Walking to school	1	0.996	0.842-1.18	0.966	0.852	0.664-1.094	0.21
Nonsmoking	1	0.915	0.721-1.161	0.463	0.774	0.556-1.076	0.127
No alcohol use	1	0.996	0.791-1.253	0.97	0.921	0.664-1.275	0.619
Never or occasionally have snacks	1	1.074	0.904-1.276	0.418	1.348	1.039-1.748	0.025^*^
Breakfast every day	1	0.945	0.785-1.138	0.552	0.879	0.669-1.156	0.357
Fresh fruit ≥4 days/week	1	1.202	0.991-1.457	0.061	0.831	0.637-1.084	0.172
Vegetables ≥4 days/week	1	1.145	0.918-1.428	0.229	1.099	0.798-1.153	0.564
Milk ≥4 days/week	1	0.886	0.748-1.05	0.162	0.823	0.64-1.059	0.13
Fizzy drinks ≥4 days/week	1	0.983	0.758-1.274	0.894	0.802	0.528-1.216	0.298
Fastfood ≥4 days/week	1	0.941	0.626-1.413	0.768	0.919	0.501-1.686	0.784
Family income ≥2000 CNY/month	1	1.045	0.856-1.275	0.666	1.442	1.045-1.99	0.026^*^
Family size ≤3	1	1.26	1.06-1.497	0.009^*^	1.144	0.886-1.477	0.302
Education of both parents above high school	1	0.943	0.641-1.385	0.764	0.774	0.422-1.418	0.406
Smoking mother	1	0.776	0.538-1.121	0.177	1.049	0.641-1.714	0.85
Smoking father	1	1.124	0.949-1.332	0.176	1.205	0.937-1.55	0.146
Unemployed mother	1	0.904	0.721-1.132	0.379	0.995	0.72-1.376	0.978
Unemployed father	1	0.963	0.71-1.307	0.81	0.954	0.609-1.495	0.837

Adjusted for age, gender and race. ^*^P < 0.05, statistically significant difference.

*BP*: blood pressure; *OR*: odds ratio; 95% CI: 95% confidence interval. *CNY*: China Yuan. 1CNY = 0.157 USD.

## Discussion

A large sample of the Chinese population was studied to explore current lifestyle health behaviors among children and adolescents. Overweight and obesity were found to be highly prevalent, particularly among the male participants. Differences in health-related factors and the recognition of weight status among participants in different weight categories were observed. Obese children were more likely to be nonsnackers, to have a shorter sleep duration, and to have a higher family income.

The high prevalence of overweight/obesity in this study was in line with many other studies [5,18-20]. It was reported that the prevalence of adolescent obesity increased dramatically from 5 to 13% in boys and from 5 to 9% in girls between 1966-70 and 1988-91 in the U.S. [21]. Data from China also indicated that the prevalence of overweight and obesity among 7–9-year-olds increased from approximately 1–2% in 1985 to 17% among girls and 25% among boys in 2000 in big cities [22]. The time trend of this rapidly growing epidemic was observed in both developed and developing countries [2-6,23]. Given its huge impact on future generations, great attention should be paid to establishing appropriate prevention and treatment programs.

In the present study, we found that obese participants were more likely to be nonsnackers compared to the normal-weight ones. Several studies have investigated the association between snacking and pediatric obesity, the results of which were conflicting. Some studies suggested that snacking might increase energy intake and thus promote weight gain [24,25], whereas others reported an inverse relationship between snacking and body weight [26-28]. The negative association found in our study may be explained by the composition of snacks and the compensation of the energy from snacks. Summerbell et al found that snack foods, compared with meals, are actually lower in fat and higher in carbohydrates [29]. It was also evidenced that among night snackers, eating cereal after the evening meal could reduce caloric intake and promote weight loss [30], indicating the important role of reasonable snack composition. In a study of snack consumption for 8 weeks, no weight gain was observed regardless of the moment of consumption and energy density of snacks [31]. This could be explained by the association between snacking and increased vigorous physical activity [26] or by rapid physical development in this period. The increased energy intake from snacking may have been compensated by activity or growth, thus limiting the development of obesity.

A substantial amount of evidence exists regarding the link between short sleep duration and childhood and adolescent overweight or obesity [28,32,33]. A meta-analysis including 36 publications found that short sleep duration was independently associated with weight gain, particularly in younger age groups [34]. The present study indicated that childhood and adolescent obesity was associated with a shorter sleep duration (<7.5 h), confirming the earlier findings. The mechanisms underlying are not fully understood. Upregulation of appetite, alterations in glucose metabolism, and decreased energy expenditure might be the potential reasons for these findings [35,36].

In our study, we found that approximately half of the parents with an overweight or obese child failed to recognize their child’s excess weight status. Approximately 35% of the parents with an obese child and 65% with an overweight child would not take measures to decrease their child’s body weight. The low rate of parental recognition of childhood overweight and obesity in this study is consistent with other studies [37-39]. This phenomenon might be due to exaggerated parental concern regarding their children. They continued to feed their babies because of worries about insufficient nutrition and energy for their children. In some cultures, big children are viewed more positively as “cute and healthy” [40], which also contributed to this situation. In addition, low recognition of excess weight status among children and adolescents was observed. These findings should be considered when planning prevention and treatment programs of pediatric obesity.

Regarding family income, Wang et al found that among children aged 6–18 years old, those in higher income groups were more likely to be obese in China and Russia, whereas those in lower income groups were at a higher risk of being obese in the U.S. [41]. Our study indicated that obese participants were more likely to have a higher family income, similar to the result found in a Jordanian population [42] and contrary to the result in Canadian children [43]. The mechanism of this region-related difference requires more exploration.

Although many studies have reported that some health-related factors, such as skipping breakfast, physical inactivity, and long hours of TV watching and video game playing, were associated with childhood and adolescent obesity [28,42,44,45], we failed to find statistically significant results, possibly due to the different definitions of the variables and an insufficient sample size.

There are limitations in the present study. First, our data were obtained from a cross-sectional study in rural Northeast China and are not representative of children and adolescents throughout the whole country. Extrapolating the conclusions to the general population should be done cautiously. Second, these analyses rely on self-reports from children and their parents, which might compromise the accuracy. Third, a relatively high drop of our population was observed. Although there were no significant differences in age and gender between the retained and excluded populations, this might still compromise the representativeness and the precision of the results. In addition, our results are based on a cross-sectional design, and thus, we could not define a causal association between health-related factors and weight status.

## Conclusions

In summary, the prevalence of pediatric overweight and obesity was relatively high, particularly among the male participants. Differences in health-related factors and the parental recognition of their children’ weight status among participants with different weight categories were observed. Obese children were more likely to be nonsnackers and to have a shorter sleep duration and higher family income. Collective evaluation of multiple health-related factors should receive more attention to better prevent and control overweight and obesity in this segment of the population.

## Abbreviations

BMI: Body mass index; BP: Blood pressure; SBP: Systolic BP; DBP: Diastolic BP; SD: Standard deviation; OR: Odds ratio; CI: Confidence interval; CNY: China Yuan.

## Competing interests

The authors declare that they have no competing interests.

## Authors’ contributions

XF Guo, LQ Zheng and Y Li participated in the protocol development, data analysis and writing of the manuscript. SS Yu, GZ Sun, HM Yang, XH Zhou and XG Zhang participated in the development of the protocol, anthropometric measurement and data collection. ZX Sun and YX Sun supervised the design and execution of the study and contributed to the revision of the manuscript. All authors read and approved the final manuscript.
